# Surgical Operations

**Published:** 1856-01

**Authors:** 


### SURGICAL OPERATIONS.

There have been two operations for Lithotomy before the class during this session, and a third was brought into the Hospital, but too far prostrated to sustain the shock of an operation. He died in a few days after admission, and afterwards two large calculi were found in the bladder. The first two cases were boys severally of the ages of four and seven years. In the first case and the eldest, a large calculus, of the mulberry variety, was removed; in the case of the second and youngest boy, the stone was small. In both the cases, recovery followed rapidly and are now entirely recovered. In these cases the bi-lateral operation was that adopted, the particulars of both of which will be reported in future by the surgeon, Prof. Hughes, himself. A number of other operations were performed before the class, including several upon the eye.

